# Estimation of energy expenditure of Nordic walking: a crossover trial

**DOI:** 10.1186/s13102-021-00240-0

**Published:** 2021-02-19

**Authors:** Sora Baek, Yuncheol Ha

**Affiliations:** 1 Department of Rehabilitation Medicine, Kangwon National University Hospital, Baengnyeong-ro 156, Chuncheon-si, Gangwon-do, 24289 Republic of Korea; 2 Department of Rehabilitation Medicine, Kangwon National University School of Medicine, Kangwondaehak-gil 1, Chuncheon-si, Gangwon-do, 24341 Republic of Korea

**Keywords:** Electromyography, Energy metabolism, Heart rate, Nordic walking, Oxygen consumption

## Abstract

**Background:**

Nordic walking (NW) requires more energy compared with conventional walking (W). However, the metabolic equation for NW has not been reported. Therefore, this study aimed to characterize responses in oxygen uptake, minute ventilation, heart rate, systolic blood pressure, and surface electromyography of the upper and lower limb muscles during NW and W and develop a metabolic equation for energy expenditure (*E*, mL·kg^− 1^·min^− 1^) of NW.

**Methods:**

This study was performed in a randomized, controlled, crossover design to test the energy expenditure during NW and W. Fifteen healthy young men were enrolled (aged 23.7 ± 3.0 years). All participants performed two randomly ordered walking tests (NW and W) on a treadmill at a predetermined stepwise incremental walking speed (3–5 km·h^− 1^) and grade (0–7%). The oxygen uptake, minute ventilation, heart rate, systolic blood pressure, and surface electromyography signals of the three upper limb muscles and three lower limb muscles in their right body were recorded and compared between NW and W using paired-t test. Multiple linear regression analysis was used to draw estimation of *E* during W and NW.

**Results:**

Oxygen uptake (+ 15.8%), minute ventilation (+ 17.0%), heart rate (+ 8.4%), and systolic blood pressure (+ 7.7%) were higher in NW than in W (*P* < .05). NW resulted in increased muscle activity in all of the upper limb muscles (*P* < .05). In the lower limb, surface electromyography activities in two of the three lower limb muscles were increased in NW than in W only during level walking (*P* < .05). Energy expenditure during W and NW was estimated as follows: *E*_*NW*_ = 6.1 + 0.09 × speed + 1.19 × speed × grade and *E*_*W*_ = 4.4 + 0.09 × speed + 1.20 × speed × grade.

**Conclusion:**

NW showed higher work intensity than W, with an oxygen consumption difference of 1.7 mL·kg^− 1^·min^− 1^. The coefficients were not different between the two walking methods. NW involved more muscles of the upper body than W.

## Background

Nordic walking (NW) is a type of walking with alternating movements of the arms and hands pushing off NW poles [1]. NW exerts beneficial effects on resting heart rate, blood pressure, exercise capacity, maximal oxygen consumption, and quality of life in patients with various diseases including diabetes mellitus, obesity, chronic obstructive pulmonary disease, and Parkinson’s disease [2]. A systematic review and meta-analysis reported that NW showed promising results in cardiovascular disorders compared to non-active control and suggested incorporation of NW for cardiovascular rehabilitation [3].

NW showed remarkable walking characteristics compared to conventional walking (W). It was known that the stride length increases and the number of steps decreases during NW than in W in the same speed. There were greater vertical oscillations of potential and kinetic energy for NW than for W [4]. For energy consumption, participants in NW consumed more energy than W in the same speed. The NW was able to improve the metabolic economy of walking reducing the cost-of-transport and increasing the optimal walking speed and self-selected walking speed [5, 6]. The use of poles actively engaged the upper body to propel the person forward during walking, resulting in higher activation of the upper body musculature [6, 7], and NW increases cardiovascular metabolism compared with W at the same walking speed [8]. These acute differences result in better movement pattern from upper limbs, increasing arm oscillations and reducing the muscle co-contraction from upper limbs [6]. When the differences in oxygen consumption between NW and W were reported as % difference, the degree of increased oxygen consumption, however, varied from 7 to 23% during flat walking [9–12]. In contrast, not proportional to the increase in energy expenditure during NW, the subjectively perceived exertion was not significantly increased [8, 11].

To our knowledge, metabolic equation of NW has not been reported. NW walking showed higher oxygen consumption compared to W, regardless of the walking speed. During uphill walking, NW also showed a greater amount of oxygen consumption than W; however, the extents of the increase in energy expenditure were reduced when walking uphill than flat walking [7, 8].

When incorporating NW in exercise training of patients, the intensity of exercise should be considered, and exact energy expenditure-estimation of NW would be required for both flat and uphill walking. Energy expenditure during exercise is estimated as the sum of the resting, horizontal, and vertical components. Regarding walking exercise, the metabolic calculation equation with walking speed (m·min^− 1^) and fractional grade was proposed as *E* (mL·kg^− 1^·min^− 1^) = 3.5 + 0.1 × speed + 1.8 × speed × fractional grade (the American College of Sports Medicine (ACSM) equation) [13]^.^

NW requires more energy than W; however, its metabolic equation has not been reported. We hypothesized that the energy consumption of Nordic walking could be derived from the horizontal and vertical values of walking. This study aimed to measure the metabolic, cardiovascular, and muscular responses during NW and W on a treadmill at incremental speed and grade. Moreover, we aimed to derive a metabolic equation for NW’s energy expenditure (*E*), which includes the horizontal and vertical elements (gait speed and slope, respectively).

## Methods

### Study design and participants

**Fig. 1:**
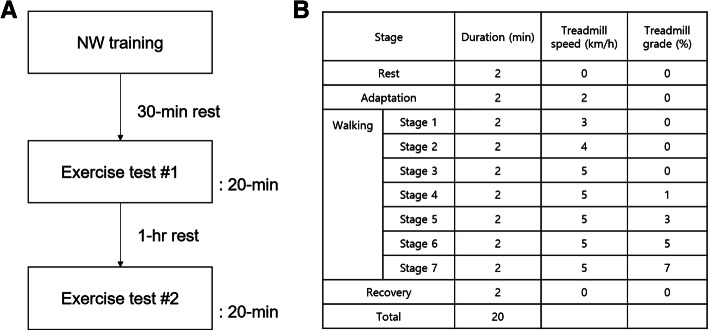
Study protocol. **a**. Flow of two random-ordered walking tests. **b**. Walking test protocol

This study was performed in a randomized, controlled, crossover design to the metabolic, cardiovascular, and muscular responses during NW and W (Fig. 1a). The experiment was conducted in a laboratory setting within 1 day, with no follow-up. Healthy adults over the age of 19 who had no Nordic pole experience were recruited as study subjects. Those with a history of arrhythmia, heart failure, or myocardial infarction or those who received treatment or medication for cardiovascular disease within the last 3 months were excluded from the study. We assumed the oxygen consumption of 18.3 mL·kg^− 1^·min^− 1^ for W and 20.5 mL·kg^− 1^·min^− 1^ for NW (standard deviation during W, 2.5 mL·kg^− 1^·min^− 1^) [11]. The estimated sample size was 11, with an alpha level of 0.05 and a beta level of 0.20. We expected a 30% dropout rate, and the final sample size was calculated as 16. The first participant was recruited on March 24, 2018. The experiment was completed by May 31, 2018, with a total of 16 people. During the experiment, 1 subject was not tested for surface electromyography; hence, the final analysis included the results of 15 subjects.

The experimental group constituted 15 healthy men. Table 1 shows the means and standard deviations for age, weight, body mass index, waist circumference, umbilicus height, resting systolic and diastolic blood pressure (SBP and DBP, respectively), resting heart rate (HR), room temperature, and room humidity. All participants had no experience with NW before this study and were fully informed about the study’s procedures and their participation’s possible risks. All voluntarily participated in the study after they provided written consent. This study was approved by the Institutional Review Board. It was conducted together with a study that analyzed the accuracy of HR measurement using a wearable band during NW [14].

**Table 1:** Characteristics of participants and laboratory room conditions (*N* = 15)

	Mean ± SD	(Min–Max)
Age (years)	23.7 ± 3.0	(19–30)
Height (cm)	174.6 ± 4.9	(167.2–182.6)
Weight (kg)	76.3 ± 13.9	(57–111.9)
Body mass index (kg/m^2^)	25.0 ± 4.1	(18.9–34.5)
Waist circumference (cm)	86.9 ± 10.7	(72–104.5)
Umbilicus height (cm)	104.9 ± 3.6	(98–113)
Resting SBP (mmHg)	124.4 ± 10.6	(110–146)
Resting DBP (mmHg)	78.9 ± 7.0	(65–88)
Resting HR (beat/min)	83.2 ± 8.3	(73–99)
Room temperature (°C)	25.9 ± 1.6	(23.4–28.2)
Room humidity (%)	33.0 ± 11.2	(21–61)

*SBP* systolic blood pressure, *DBP* diastolic blood pressure, *HR* heart rate

### NW technique

Participants visited the exercise testing laboratory on the day of the graded walking tests. Participants were sent a homepage link (http://www.nordicwalking.or.kr) about the Nordic walking method in advance to the experimental day. On experiment day, they underwent a 1-h training on holding and releasing the Nordic pole and walked with Nordic pole on surface and on the treadmill with a NW instructor from the Korean Nordic Walking Federation/Korean Walking Association (Y. Ha) before the walking test. The NW technique emerged from a training modality that is typical of cross-country skiing, requiring a specific technique: moving the extended arms similar to the range of movement of natural walking, maintaining the upper body upright, maintaining a backward pole position during the loading phase, using the poles actively and dynamically, and controlling the poles by hands gripping with grasp/release patterns [15]. Each participant’s pole length (Nordic Friend, Gabel, Italy) was set at the umbilicus height. The weight of the walking pole was 196 g. After familiarization with NW on a treadmill, they were asked to rest for at least 30 min before the start of the walking test.

### Graded walking test protocol

The graded walking test protocol on the treadmill constituted 20-min stages of rest, adaptation, walking, and recovery. The participants warmed up and familiarized for 2 min at 2 km·h^− 1^, the same speed with the first walking test stage. The walking tests constituted seven stages, with a 2-min duration for each stage: walking on a treadmill (STEX 8100TD; Taeha Mechatronics, Anyang-Si, Korea) at 3 km·h^− 1^, 4 km·h^− 1^, and 5 km·h^− 1^ at 0% inclination and 1, 3, 5, and 7% inclination at 5 km·h^− 1^ (Fig. 1b).

The participants walked on the treadmill at a stepwise incremental speed and grade. Two walking conditions (NW and W) were used with a randomized sequence using a random number. The blood pressure cuff was worn on the left upper arm, and SBP/DBP were measured at the end of each stage. HR was assessed in 30-s intervals using 12-lead electrocardiography (Philips StressVue, Philips, the Netherlands), and the highest HR value was selected for each walking stage. Exertion was also rated at each walking stage; they were rated using the 6–20-point Borg rating of perceived exertion (RPE) scale [16].

### Ventilatory gas analysis

The oxygen consumption (*V̇*O_2_), carbon dioxide production (*V̇*CO_2_), expired ventilation per minute (*V̇*E), respiratory exchange ratio (RER), and respiratory rate (RR) were measured using a ventilatory gas analysis system (Ultima PFX®, MGC Diagnostics Corporation, St Paul, MN, USA). The measured values during the 2 min of each walking stage were averaged.

### Surface electromyography

The surface electromyographic signals of the mid deltoid (DEL, the midpoint between the acromion and the deltoid tubercle), biceps brachii (BB, the thickest muscle belly of BB), triceps brachii (TB, the midpoint between the acromion and the olecranon), vastus lateralis (VL, five finger’s breadth upward and lateral from the patella), medial gastrocnemius (GCM, the medial belly of the calf muscle), and tibialis anterior (TA, four finger’s breadth downward from the tibial tuberosity) in their right body were recorded using Model 586 Desktop DTS Receiver and Model 542 Desktop DTS EMG Sensor (Noraxon USA, Inc., Scottsdale, AZ).

Before applying the surface electrodes, the skin was cleaned with an alcohol swab to reduce impedance. A skilled physiotherapist attached all electrodes to the skin on the midpoint of the contracted muscle belly parallel to the muscle fibers with an adhesive tape. The sampling frequency was 1500 Hz. The data were band-pass filtered with a 10–250 Hz and rectified in the acquisition software (myoRESEARCH® 3). The root mean square value was acquired for each walking stage.

### Statistical analysis

The acquired data were analyzed using the SPSS ver. 23 (IBM, Armonk, NY). Hemodynamic responses, ventilatory gas analysis, and surface electromyographic results were compared between NW and W using the paired t-test. A *P*-value < 0.05 was considered significant.

Simple and multiple linear regression analyses were conducted to estimate *E* for NW. The *V̇O*_*2*_ (mL·kg^− 1^·min^− 1^) with different walking speeds at flat walking (walking stage 1 to 3; grade = 0%; speed 3 to 5 km·h^− 1^ or 50 to 83 m·min^− 1^) was analyzed using a simple linear regression analysis. The *V̇O*_*2*_ in different grades with constant speed (walking stage 3 to 7; speed = 5 km·h^− 1^ or 83 m·min^− 1^) was estimated using a simple linear regression analysis. Multiple linear regression analysis was used for walking stage 1 to 7 for treadmill speed and the interaction of treadmill speed and grade.

*E* for W also estimated with simple and multiple linear regression analyses in the same way as NW.

## Results

### Ventilatory gas analysis, HR, and blood pressure during graded NW and W

**Fig. 2:**
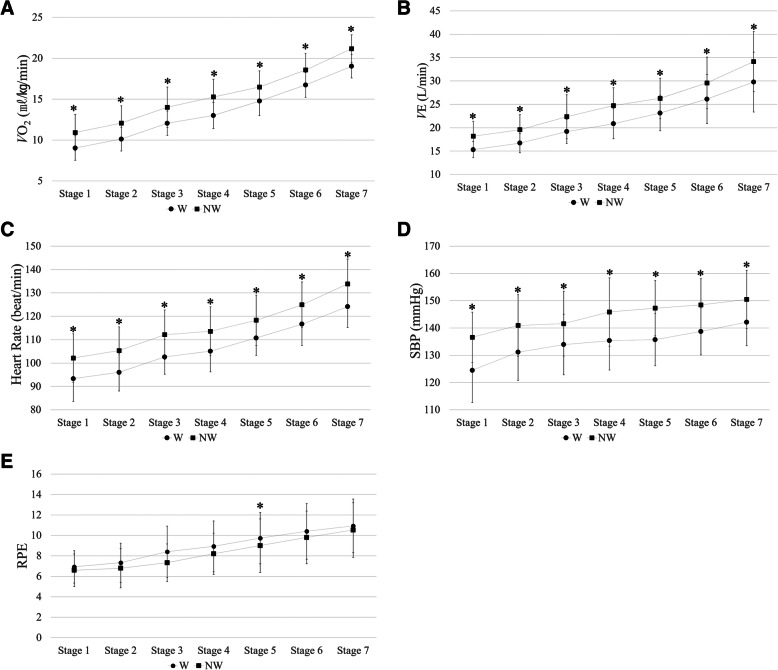
Ventilatory gas analysis results during walking (W) and Nordic walking (NW). **a**. Oxygen uptake (*V̇*O2). **b**. Ventilation (*V̇*E). **c**. Heart rate. **d**. Systolic blood pressure (SBP). **e**. Rate of perceived exertion (RPE). * P < .05

Ventilatory gas analysis results showed increased metabolism and hemodynamic responses in NW than in W. The *V̇O*_*2*_ (+ 15.8%), *V̇CO*_*2*_ (+ 17.0%), *V̇E* (+ 17.0%), RR (+ 18.2%), SBP (+ 7.7%), DBP (+ 6.9%), and HR (+ 8.4%) were significantly higher in NW than in W (*P*-value < 0.05) (Table 2). *V̇*O_2_, *V̇*E, HR, and SBP showed significant differences between NW and W in all walking stages (Fig. 2). Conversely, RPE was less in NW than in W (Table 2). Chest pain was absent in both NW and W. The degree of difficulty in breathing was very low, with no significant difference between NW and W.

**Table 2:** Ventilatory gas analysis and surface electromyographic results during Nordic walking (NW) and conventional walking (W)

	NW	W	*P*-value	Δ (NW–W)	Δ (NW–W)/W (%)
*V̇*O_2_ (mL·kg^−1^·min^− 1^)	15.5 ± 3.9	13.5 ± 3.7	<.01	2.0 ± 1.6	15.8
*V̇*CO_2_ (mL·min^− 1^)	968.9 ± 300.0	840.3 ± 281.2	<.01	128.6 ± 111.4	17.0
*V̇*E (L·min^− 1^)	25.0 ± 6.9	21.6 ± 6.1	<.01	3.4 ± 3.3	17.0
RER	0.823 ± 0.058	0.815 ± 0.069	<.05	0.007 ± 0.035	1.1
RR (beat·min^− 1^)	24.6 ± 3.5	21.0 ± 3.1	<.01	3.5 ± 3.8	18.2
SBP (mmHg)	144.5 ± 11.5	134.5 ± 11.2	<.01	10 ± 9.1	7.7
DBP (mmHg)	79.2 ± 5.5	74.4 ± 5.1	<.01	4.9 ± 5.9	6.9
HR (beat·min^− 1^)	115.8 ± 14.4	106.9 ± 13.2	<.01	8.8 ± 5.6	8.4
RPE	8.3 ± 2.6	9.0 ± 2.7	<.01	− 0.6 ± 1.4	−5.9
DEL (μV)	12.0 ± 4.8	6.7 ± 3.7	<.01	5.3 ± 3.1	94.9
BB (μV)	18.9 ± 7.2	5.6 ± 4.5	<.01	13.3 ± 8.8	381.4
TB (μV)	21.8 ± 10.1	6.4 ± 3.2	<.01	15.4 ± 9.2	308.6
VL (μV)	65.4 ± 57.7	67.6 ± 57.9	.60	−2.2 ± 39.1	34.7
TA (μV)	45.7 ± 14.4	40.9 ± 11.3	<.01	4.8 ± 9.8	13.3
GCM (μV)	39.7 ± 11.8	36.5 ± 12.3	<.01	3.3 ± 6.8	11.7

*RER* respiratory exchange ratio, *RR* respiratory rate, *SBP* systolic blood pressure, *DBD* diastolic blood pressure, *HR* heart rate, *RPE* rating of perceived exertion, *DEL* deltoid, *BB* biceps brachii, *TB* triceps brachii, *VL* vastus lateralis, *TA* tibialis anterior, *GCM* gastrocnemius, *L3* L3 paraspinals

### Activation of the upper and lower limb muscles during graded NW and W

**Fig. 3:**
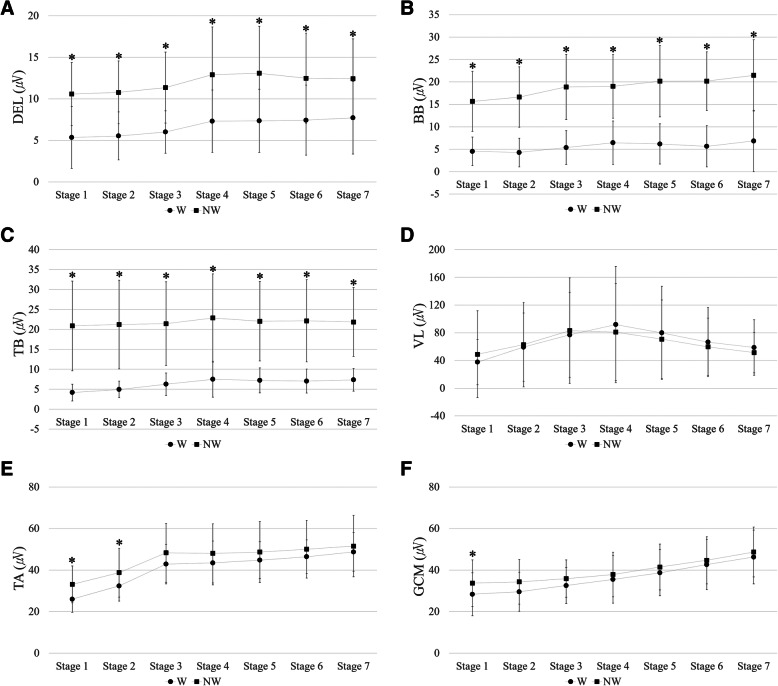
Surface electromyographic results of the upper and lower limb muscles during conventional walking (W) and Nordic walking (NW). **a** Lateral deltoid (DEL). **b** Biceps brachii (BB). **c** Triceps brachii (TB). **d** Vastus lateralis (VL). **e** Tibialis anterior (TA). **f** Gastrocnemius (GCM). * P < .05

The differences in muscle activities between W and NW varied between the upper and lower extremities. The upper limb muscle activities (DEL, BB, and TB) were significantly higher in NW than in W (*P*-value < 0.05) (Table 2). These differences were significant in all walking stages (Fig. 3). No significant difference was found in the activity of VL between the two walking conditions (Table 2) in lower extremity muscles. TA and GCM activities were higher in NW (Table 3); however, it showed a significant difference only in level walking (stages 1 and 2 for TA; stage 1 for GCM) (Fig. 3).

**Fig. 4:**
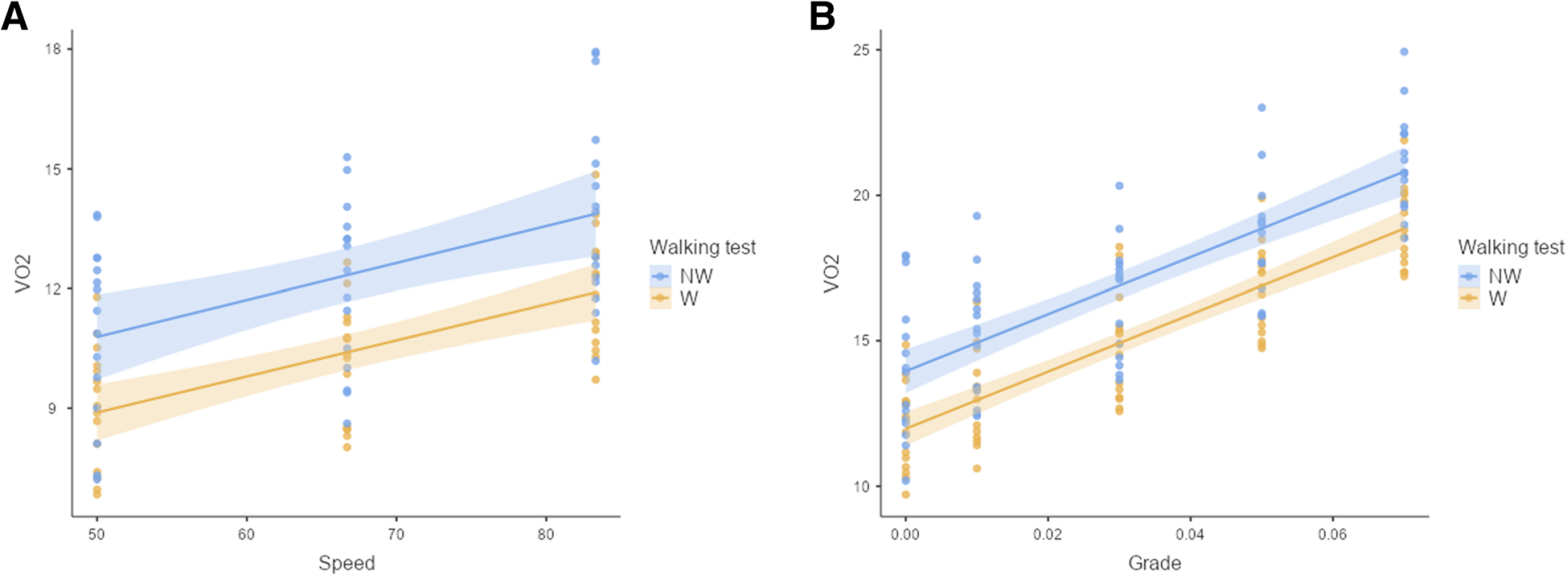
Scatter plots of *V*O_2_ (regression line with standard error) in different walking speeds (**a**) and grades (**b**) in Nordic walking (NW) and conventional walking (W)

**Table 3:** Oxygen consumption (*V̇*O_2_, mL·kg^− 1^·min^− 1^) at each walking stage during Nordic walking (NW) and conventional walking (W)

Walking stage	Treadmill speed (km·h^− 1^)	Treadmill grade (%)	*V̇*O_2_ NW	*V̇*O_2_ W	Δ*V̇*O_2_ (%*V̇*O_2_)	*P*-value
Stage 1	3	0	10.9 ± 2.2	9.0 ± 1.5	1.9 (20.9)	<.01
Stage 2	4	0	12.1 ± 2.1	10.1 ± 1.5	2.0 (19.3)	<.01
Stage 3	5	0	14.0 ± 2.5	12.0 ± 1.5	2.0 (16.2)	<.01
Stage 4	5	1	15.3 ± 2.1	13.0 ± 1.6	2.3 (17.4)	<.01
Stage 5	5	3	16.5 ± 2.0	14.8 ± 1.8	1.7 (11.6)	<.01
Stage 6	5	5	18.6 ± 2.0	16.7 ± 1.5	1.8 (10.9)	<.01
Stage 7	5	7	21.2 ± 1.7	19.0 ± 1.4	2.1 (11.3)	<.01

Δ*V̇*O_2_ = *V̇*O_2_NW – *V̇*O_2_W

%*V̇*O_2_ = (*V̇*O_2_NW – *V̇*O_2_W) / *V̇*O_2_W × 100

### Estimation of oxygen consumption of NW and W

Ventilatory gas analysis results during NW and W showed that the oxygen uptake was 10.9 ± 2.2 to 21.2 ± 1.7 mL·kg^− 1^·min^− 1^ during NW and 9.0 ± 1.5 to 19.0 ± 1.4 mL·kg^− 1^·min^− 1^ during W. The difference between NW and W regarding oxygen consumption from stage 1 to 7 ranged 1.7–2.3 mL·kg^− 1^·min^− 1^ (Table 3).

The *E* or *V̇O*_*2*_ (mL·kg^− 1^·min^− 1^) during NW with different walking speeds at flat walking (walking stage 1 to 3; grade = 0%; speed 3 to 5 km·h^− 1^ or 50 to 83 m·min^− 1^) was analyzed using a simple linear regression analysis, and coefficients for walking speed were estimated (Table 4, Fig. 4a). Speed is expressed in m·min^− 1^, and fractional grade is grade percentage expressed in decimal formal (e.g., 10% = 0.10):

$$ {E}_{NW}=6.3+0.09\times \mathrm{speed} $$

**Table 4:** Estimation of oxygen consumption (*V̇*O_2_, mL·kg^−1^·min^−1^) during Nordic walking (NW) and conventional walking (W) using simple linear regression analysis

	**Walking stage (treadmill grade = 0.00)**					**Linear regression analysis**			
	Stage 1	Stage 2		Stage 3		Constant	Coefficients for speed	R	R^2^
Treadmill speed	50.0 m·min^−1^ (3 km·h^− 1^)	66.7 m·min^− 1^ (4 km·h^− 1^)		83.3 m·min^− 1^ (5 km·h^− 1^)					
*V̇*O_2_ NW	10.9 ± 0.57	12.1 ± 0.54		14.0 ± 0.64		6.16	0.09	0.497	0.247
*V̇*O_2_ W	9.0 ± 0.38	10.1 ± 0.38		12.0 ± 0.38		4.36	0.09	0.648	0.420
	**Walking stage (treadmill speed = 83.3 m·min**^**− 1**^**)**					**Linear regression analysis**			
	Stage 3	Stage 4	Stage 5	Stage 6	Stage 7	Constant	Coefficients for grade	R	R^2^
Treadmill grade	0.00	0.01	0.03	0.05	0.07				
*V̇*O2 NW	14.0 ± 0.64	15.3 ± 0.54	16.5 ± 0.51	18.6 ± 0.52	21.2 ± 0.44	13.9	99.2	0.757	0.573
*V̇*O2 W	12.0 ± 0.38	13.0 ± 0.43	14.8 ± 0.47	16.7 ± 0.39	19.0 ± 0.37	11.9	100.1	0.821	0.675

Values are mean ± standard error

Oxygen consumption during NW in different grades with constant speed (walking stage 3 to 7; speed = 5 km·h^− 1^ or 83 m·min^− 1^) was estimated using a simple linear regression analysis, and coefficients for fractional grade were estimated (Table 4, Fig. 4b):

$$ {E}_{NW}=13.9+99.2\times \mathrm{fractional}\ \mathrm{grade} $$

Coefficient 0.09 for speed and speed value (83.3 m·min^− 1^) were integrated to the equation:

$$ {\displaystyle \begin{array}{c}{E}_{NW}=\left(13.9-7.5\right)+7.5+\left(99.2/83.3\right)\times 83.3\times \mathrm{fractional}\ \mathrm{grade}\\ {}=6.4+0.09\times 83.3\ \mathrm{m}\cdotp {\min}^{-1}+1.19\times 83.3\ \mathrm{m}\cdotp {\min}^{-1}\times \mathrm{fractional}\ \mathrm{grade}\end{array}} $$

Simple linear regression analyses were conducted to estimate *E* for W in the same way as NW.

$$ {\displaystyle \begin{array}{c}{E}_W=4.4+0.09\times \mathrm{speed}\ \left(\mathrm{walking}\ \mathrm{stage}\ 1\ \mathrm{to}\ 3\right)\\ {}{E}_W=11.9+100.1\times \mathrm{fractional}\ \mathrm{grade}\ \left(\mathrm{walking}\ \mathrm{stage}\ 3\ \mathrm{to}\ 7\right)\\ {}\begin{array}{c}{E}_W=\left(11.9-7.5\right)+7.5+\left(100.1/83.3\right)\times 83.3\times \mathrm{fractional}\ \mathrm{grade}\\ {}=4.4+0.09\times 83.3\ \mathrm{m}\cdotp {\min}^{-1}+1.20\times 83.3\ \mathrm{m}\cdotp {\min}^{-1}\times \mathrm{fractional}\ \mathrm{grade}\end{array}\end{array}} $$

From the estimation equations from the two-step simple linear regression analysis, the constant values was 6.4 for NW and 4.4 for W, and the difference was 2.0 mL·kg^− 1^·min^− 1^. Coefficients for speed were 0.09 in both NW and W. Coefficients for fractional grade were 1.19 for NW and 1.20 for W.

As a result of multiple linear regressions for speed and speed × fractional grade, the final formulae for NW and W is shown below (Table 5):

$$ {E}_{NW}\left(\mathrm{m}\mathrm{L}\cdotp {\mathrm{kg}}^{-1}\cdotp {\min}^{-1}\right)=6.1+0.09\times \mathrm{speed}\ \left(\mathrm{m}\cdotp {\min}^{-1}\right)+1.19\times \mathrm{speed}\times \mathrm{fractional}\ \mathrm{grade} $$

$$ {E}_W\left(\mathrm{m}\mathrm{L}\cdotp {\mathrm{kg}}^{-1}\cdotp {\min}^{-1}\right)=4.4+0.09\times \mathrm{speed}\ \left(\mathrm{m}\cdotp {\min}^{-1}\right)+1.20\times \mathrm{speed}\times \mathrm{fractional}\ \mathrm{grade} $$

**Table 5:** Estimation of oxygen consumption (*V̇*O_2_, mL·kg^−1^·min^−1^) during Nordic walking (NW) and conventional walking (W) using multiple linear regression analysis

		Model coefficients			Overall model fit			
		Estimate	t	***P***-value	R	R^**2**^	Adjusted R^**2**^	***P***-value
*V̇*O_2_ NW	Constant	6.14	4.38	< 0.001	0.852	0.725	0.720	< 0.001
	Speed	0.09	4.78	< 0.001				
	Speed × grade	1.19	10.97	< 0.001				
*V̇*O_2_ W	Constant	4.39	4.24	< 0.001	0.911	0.829	0.826	< 0.001
	Speed	0.09	6.26	< 0.001				
	Speed × grade	1.20	14.88	< 0.001				

Both final formulae were statistically significant, explaining 72.0% of the relationship between *V̇*O_2_-NW and speed, speed × fractional grade, and 82.6% of the relationship between *V̇*O_2_-W and speed, speed × fractional grade. The constant value in the final equation for W was 4.4; this was somewhat higher than the known resting oxygen consumption (3.5 mL·kg^− 1^·min^− 1^) [13]^.^ The coefficient for speed was 0.09, which was similar to the known value of 0.1 [13]^.^ However, the coefficient for grade × speed was 1.2, which was less than the known value of 1.8 [13]^.^ In the above estimation, NW required more oxygen consumption than W, and the difference of constants of the two formulae was 1.7 mL·kg^− 1^·min^− 1^ in multiple linear regression analysis. The coefficients for speed and speed × fractional grade were not different between the two walking methods.

## Discussion

To the best of our knowledge, this is the first study that has derived the energy consumption of Nordic walking through the horizontal and vertical values of walking. When walking at the given speed and slope, NW had higher exercise intensity than W. The *V̇O*_*2*_ (+ 15.8%), *V̇CO*_*2*_ (+ 17.0%), *V̇E* (+ 17.0%), RR (+ 18.2%), SBP (+ 7.7%), DBP (+ 6.9%), and HR (+ 8.4%) were significantly higher in NW than in W in all walking stages. In this study, we derived new energy estimation equations of NW and W from the measured *V̇*O_2_ and walking speed and slope: *E*_*W*_ = 4.4 + 0.09 × speed (m·min^− 1^) + 1.20 × speed × fractional grade and *E*_*NW*_ = 6.1 + 0.09 × speed (m·min^− 1^) + 1.19 × speed × fractional grade. The constant value in the final equation for W was 4.4 mL·kg^− 1^·min^− 1^, which is somewhat higher than the ACSM equation’s known resting oxygen consumption of 3.5 mL·kg^− 1^·min^− 1^ [13]^.^ The coefficient for speed was 0.09, which is similar to the known value of 0.1 [13].

Previous studies reported the energy consumption of NW as a percent difference (%*V̇*O_2_) compared to the consumption of W. The result varied depending on the walking speed and the slope from 7 to 23% during flat walking [9–12]. For uphill walking, Figard-Fabre et al. [8] measured oxygen consumption at 0 and 5% grade when walking at 4 km·h^− 1^, and the %*V̇*O_2_ was 16% at 0% grade and reduced to 12% at 5% grade. In our study, %*V̇*O_2_ was highest in minimum energy expenditure and lowest in maximum energy consumption. Increasing the walking speed from 3 km/hr. to 5 km/hr., the %*V̇*O2 of NW compared to W decreased from 20.9 to 16.2% of W. As the slope of the uphill increased (from 0 to 7%) at a constant speed (5 km·h^− 1^), %*V̇*O2 decreased from the highest (17.4%) to the lowest (10.9%). Conversely, the difference (Δ*V̇*O_2_) between NW and W was rather constant than proportional. The difference between constants of the two formulae for NW and W was 1.7 mL·kg^− 1^·min^− 1^ in multiple linear regression analysis derived from speed and speed × grade. The results of a study by Figard-Fabre et al. [8] were consistent with the values in our eq. (4 km·h^− 1^, 0%, 10.4 mL·kg^− 1^·min^− 1^ for W; 12.1 mL·kg^− 1^·min^− 1^ for NW; and 16% for %*V̇*O_2_; 4 km·h^− 1^, 5%, 14.4 mL·kg^− 1^·min^− 1^ for W; 16.1 mL·kg^− 1^·min^− 1^ for NW; and 12% for %*V̇*O_2_). Pellegrini et al. [7] measured oxygen consumption at 15% grade when walking at 4 km·h^− 1^, and %*V̇*O_2_ was 6.9%. This value was consistent with that of our eq. (4 km·h^− 1^, 15%, 22.4 mL·kg^− 1^·min^− 1^ for W; 24 mL·kg^− 1^·min^− 1^ for NW; 7% for %*V̇*O_2_).

The coefficient for walking speed × grade was 1.20 in our study and smaller than ACSM’s coefficient. The ACSM regression equations developed to estimate oxygen uptake have known limitations that lead to overestimation of energy expenditure, particularly at higher work rates [17]^.^ Kokkinos et al. recently developed a new energy equation for walking from the Fitness Registry and the Importance of Exercise National Database (FRIEND) and suggested small cofficient value of 0.79 for walking speed × grade: *E* (mL·kg^− 1^·min^− 1^) = 3.5 + 0.17 × speed (m·min^− 1^) + 0.79 × speed × fractional grade (FRIEND equation) [17].

Pellegrini et al. reported that higher total mechanical work for NW mainly due to the greater work required to move the upper limbs and poles [4]. The increase in exercise intensity and oxygen consumption by NW is due to the increased upper limb muscle activity. The upper extremity’s increased activity was identified through the surface EMG signals. Walking with upper body exercise [18] can increase the *V̇*O_2_. The activities of the upper limb muscles (DEL, BB, and TB) were significantly higher in NW than in W. No significant differences were found between the two walking conditions in the lower extremity muscles, except the TA and GCM muscles during slow level walking. Regarding walking while exercising the upper limbs, the amount of oxygen consumption significantly increased compared with normal walking [18]. The increase in oxygen consumption at NW seems to be related to increase in the activity of the upper limb muscles. This result is in line with previous findings [6, 7].

Interestingly, oxygen consumption increased significantly in NW compared with W; however, the subjective difficulty, the RPE, did not show a significant difference between NW and W at level walking and rather lower RPE with NW during uphill walking. Previous studies showed higher RPE [9], lower RPE [11], or no difference [8] with pole walking compared with W. During uphill walking, decreased RPE with NW was reported in previous studies with pole walking [8, 19]. According to a study comparing the RPE of NW and W on downhill, uphill, and level walking, the RPE in NW on uphill walking was significantly decreased compared with W [8].

The formula for oxygen consumption in NW was derived only from a non-fast walking speed (3–5 km·h^− 1^) and a slope within 7%. Energy estimation may differ from the predicted values in a faster or higher slope. In addition, NW energy consumption could be influenced by several factors other than walking speed and slope grade. When a relatively short pole was used, energy consumption was increased in the uphill than in the case of a normal length pole [20]. By contrast, pole weight does not appear to have a significant effect on energy consumption [21]. As a result of comparing W and four different types of NW, muscle activity and metabolic response were different according to the type of NW; however, all types of NW showed higher metabolic response and muscle activity than W [22]^.^

NW is usually conducted outdoors. We measured indoors on a treadmill. Oxygen consumption results of studies conducted in an environment other than the treadmill (outdoor field study) showed a difference from the predicted value of our formula [10, 12]^.^ Church et al. [10] reported that the average amounts of oxygen consumption were 13.9 mL·kg^− 1^·min^− 1^ during W and 16.7 mL·kg^− 1^·min^− 1^ during NW at average self-selected walking speeds (5.6 km·h^− 1^ for male and 5.9 km·h^− 1^ for female participants). The values were larger than the estimated *V̇*O_2_ values derived from our eq. (12.8 and 13.3 mL·kg^− 1^·min^− 1^ for W and 14.5 and 15.0 mL·kg^− 1^·min^− 1^ for NW). The oxygen consumption values measured in the field study [10] were greater than our predicted values; particularly, the *V̇*O_2_ during NW was higher than the predicted value. Regarding the field test, terrain characteristics would differ from those of a treadmill, potentially causing a difference in poling force or muscle activity. The difference in walking terrain leads to a difference in oxygen consumption during NW [23]. According to a study by Schiffer et al. [23], the oxygen consumption during NW was significantly increased in a naturally grown soccer lawn than concrete.

This study had limitations. We included only young healthy subjects without cardiovascular or neuromuscular disorders; however, the potential target group for therapeutic application of Nordic gait is thought to be the elderly and disables [3, 24]. The participants of our study were all beginners with no Nordic pole experience and all were young males. The proficiency of NW methods can affect gait technique; however, even after a 4-week NW training three times a week, the difference in oxygen consumption between NW and W did not significantly change before and after the training [8]. Moreover, the activation pattern of the surface electromyography of our results was similar to that of the NW-skilled participants. Surface electromyography analysis in participants who were experienced in NW showed that the activities of the BB, TB, and deltoid anterior in NW were significantly increased compared with those in W, which was significant in both level and uphill [7]. Furthermore, the activities of the lower extremity muscles, such as the TA, gastrocnemius lateralis, and VL did not show a significant difference in NW and W, which was the same in level and uphill [7]. These muscle activation patterns are similar to those found in our study. According to a previous study, the changes in the aspects of respiratory gas analysis did not differ by sex [9].

We suggest a cautious application of the derived formula to a range other than 3–5 km/hr. and grade of 0–7%. In field walking, it should be considered that more energy can be consumed than in a treadmill. We derive the formula only for young men, and we believe that further analysis in women and the elderly is necessary.

## Conclusions

We developed an equation to estimate oxygen consumption during NW, which was constantly consuming more oxygen by 1.7 mL·kg^− 1^·min^− 1^. Estimation of oxygen consumption during NW in faster speed is necessary for more precise exercise prescription. The difference in *V̇*O_2_ between NW and W was constant both in slow and fast walking speeds and both in level and uphill grades.

## Data Availability

The datasets used and/or analyzed during the current study are available from the corresponding author on reasonable request.

## Abbreviations

NW: Nordic walking
W: Conventional walking
*V̇*O_2_: Oxygen uptake
*V̇*E: Minute ventilation
RER: Respiratory exchange ratio
RR: Respiratory rate
DEL: Mid deltoid
BB: Biceps brachii
TB: Triceps brachii
VL: Vastus lateralis
GCM: Medial gastrocnemius
TA: Tibialis anterior
HR: Heart rate
SBP: Systolic blood pressure
DBP: Diastolic blood pressure
sEMG: Surface electromyography
*E*: Energy expenditure
ACSM: American College of Sports Medicine
RPE: Rating of perceived exertion
